# Supplementary material for: CIPK‐B is essential for salt stress signalling in Marchantia polymorpha
Source: New Phytol. 2023 Jan 20;237(6):2210–23. doi: 10.1111/nph.18633 (PMC10953335; doi:10.1111/nph.18633)
Supplement: Supplementary file 1 — Fig. S1 Conservation of key domains in Marchantia polymorpha CBLs. Fig. S2 Conservation of key domains in Marchantia polymorpha CIPKs. Fig. S3 Regulation of CBL and CIPK gene expression by salt stress in Tak‐1 plants. Fig. S4 Western blotting confirms expression of all Marchantia polymorpha CBLs and CIPKs in strains used for yeast two‐hybrid assays. Fig. S5 Bimolecular fluorescence complementation confirms all Marchantia polymorpha CBLs and CIPKS interact with Nicotiana benthamiana. Fig. S6 Both cipk‐b knockout mutant lines encode proteins with premature stop codons. Table S1 Gene ID numbers for sequences used in bioinformatics in this study. Table S2 Primers used for RT‐qPCR in this study. Table S3 Final constructs created and used in this study. Please note: Wiley is not responsible for the content or functionality of any Supporting Information supplied by the authors. Any queries (other than missing material) should be directed to the New Phytologist Central Office. [file NPH-237-2210-s001.pdf]

New Phytologist Supporting Information

Article title: **CIPK-B is essential for salt stress signalling in *Marchantia polymorpha***

Authors: Connor Tansley[1], James Houghton[1*], Althea M. E. Rose[1*], Bartosz Witek[1], Rocky D. Payet[1], Taoyang Wu[2], J. Benjamin Miller[1]

Article acceptance date: 13 November 2022

The following Supporting Information is available for this article:

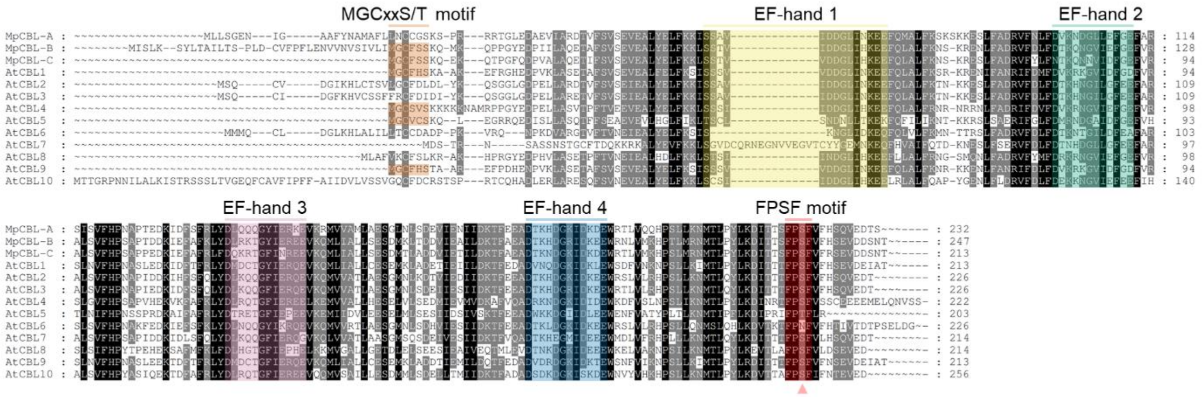

Fig. S1: Conservation of key domains in *Marchantia polymorpha* CBLs. Sequence alignment of CBL proteins from *A. thaliana* and *M. polymorpha*, with black/grey shading denoting the level of conservation. All three *M. polymorpha* CBLs have four conserved EF-hands (as indicated), including the first EF-hand containing a loop region of 14 amino acids. MpCBL-B/C (but MpCBL-A) contain the MGCxxS/T myristoylation and palmitoylation motif for localisation to the plasma membrane. All three *M. polymorpha* CBLs also have the conserved FPSF motif, including the conserved serine residue that can be regulated by phosphorylation (arrow head).

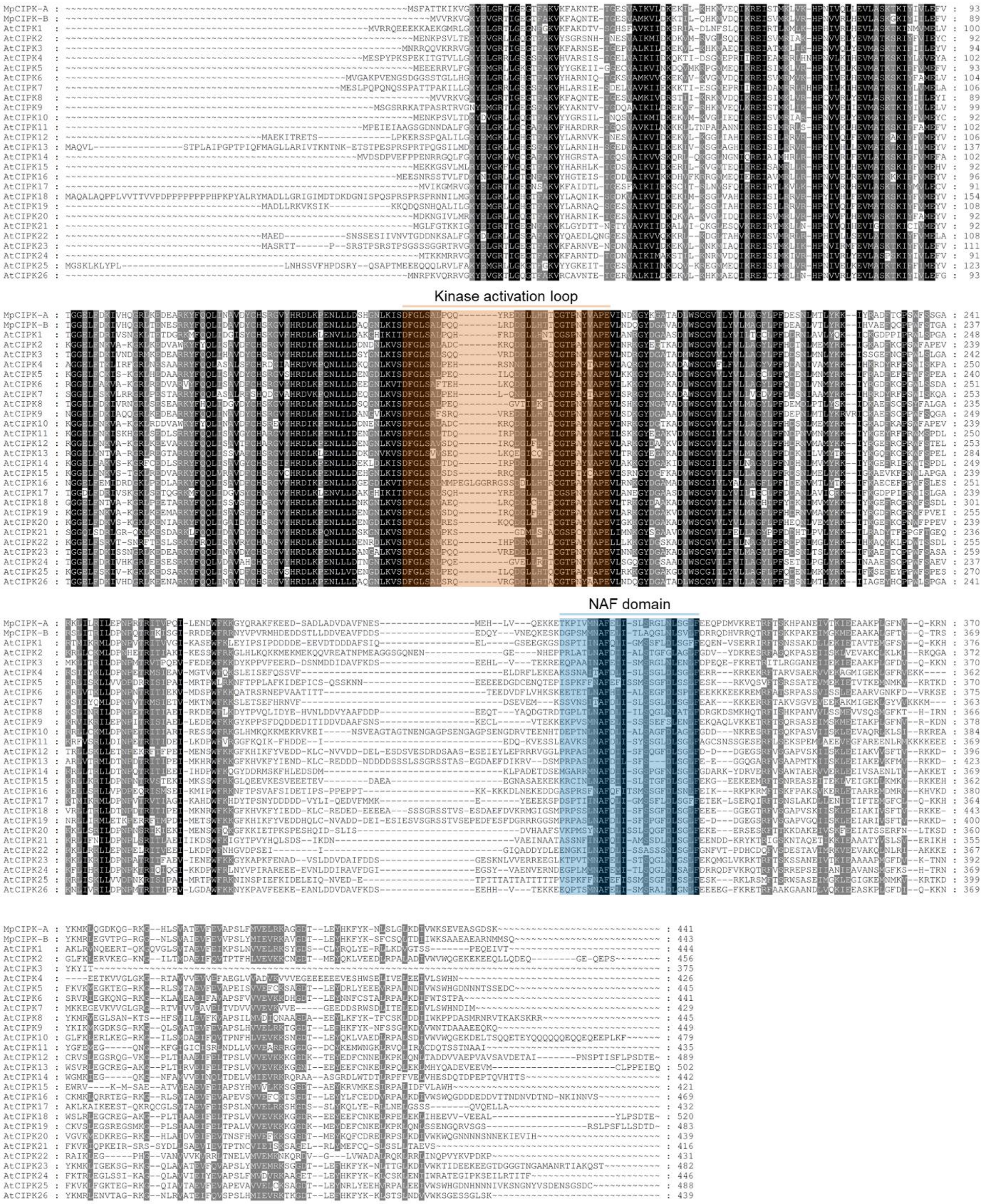

Fig. S2: Conservation of key domains in *Marchantia polymorpha* CIPKs. Sequence alignment of CIPK proteins from *A. thaliana* and *M. polymorpha*, with black/grey shading denoting the level of conservation. Both *M. polymorpha* CIPKs have conserved kinase domains, with an activation loop (defined between the DFG and APE residues), and conserved NAF domains (as indicated).

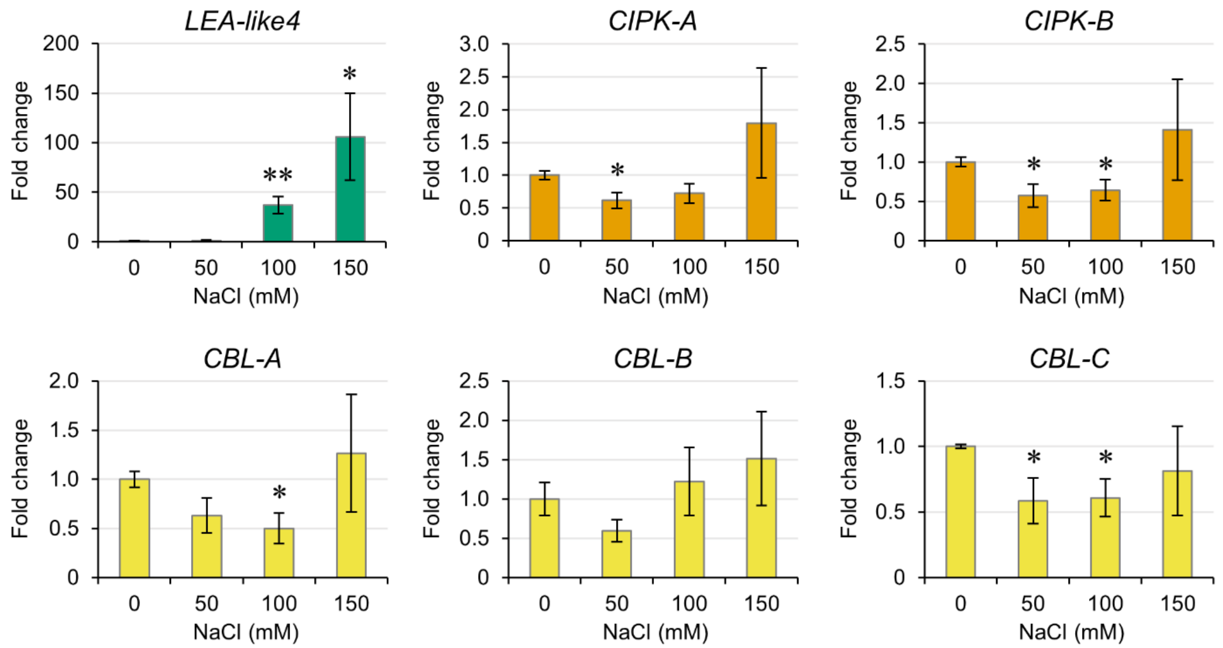

Fig. S3: Regulation of *CBL* and *CIPK* gene expression by salt stress in Tak-1 plants. Expression changes of the indicated genes were measured by RT-qPCR in thallus tissue of Tak-1 plants grown in the presence of the indicated concentrations of NaCl for 7 days. Data represent mean ± one standard error from three independent biological replicates. Significance in a pairwise two-tailed t-test relative to 0 mM treatment is indicated at $p<0.05$ (one star) or $p<0.01$ (two stars).

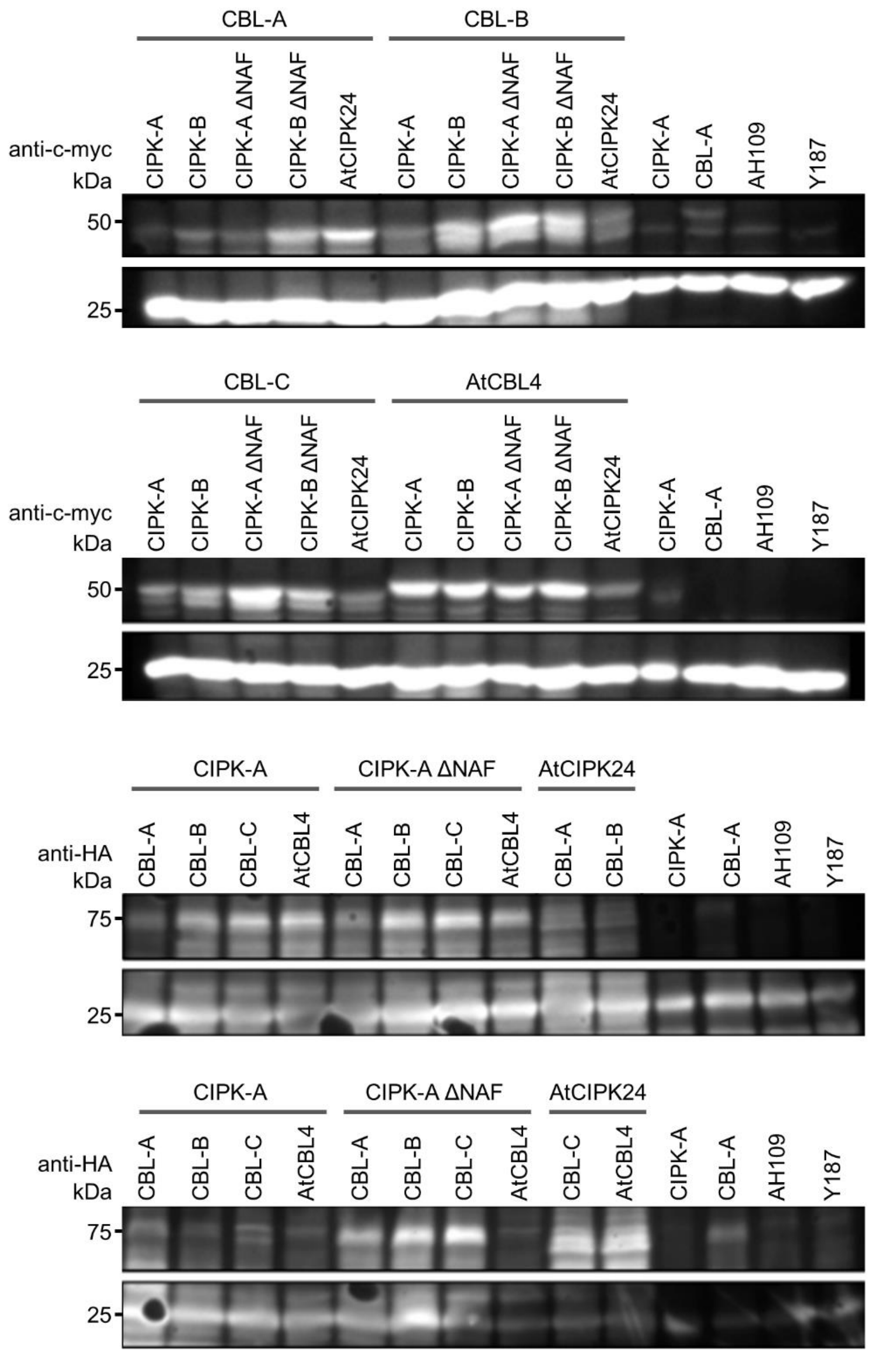

Fig. S4: Western blotting confirms expression of all *Marchantia polymorpha* CBLs and CIPKs in strains used for yeast two-hybrid assays. Western blotting using c-myc and HA antibodies identified proteins of the expected size for GAL4-BD-3xc-myc-CBLs and GAL4-AD-3xHA-CIPKs, respectively (upper panels). Expected sizes of the GAL4-BD-3xc-myc-CBLs are: 48.5 (MpCBL-A), 46.6 (MpCBL-B and MpCBL-C) and 47.8 kDa (AtCBL4). Expected sizes of the GAL4-AD-3xHA-CIPKs are: 69.7 (MpCIPK-A), 70.0 (MpCIPK-B), 67.1 (MpCIPK-A ΔNAF), 67.4 (MpCIPK-B ΔNAF) and 70.2 kDa (AtCIPK24). Lower panels correspond to loading control, using native yeast peroxidase (PRX1) activity as a proxy for total protein (expected size of 25 kDa).

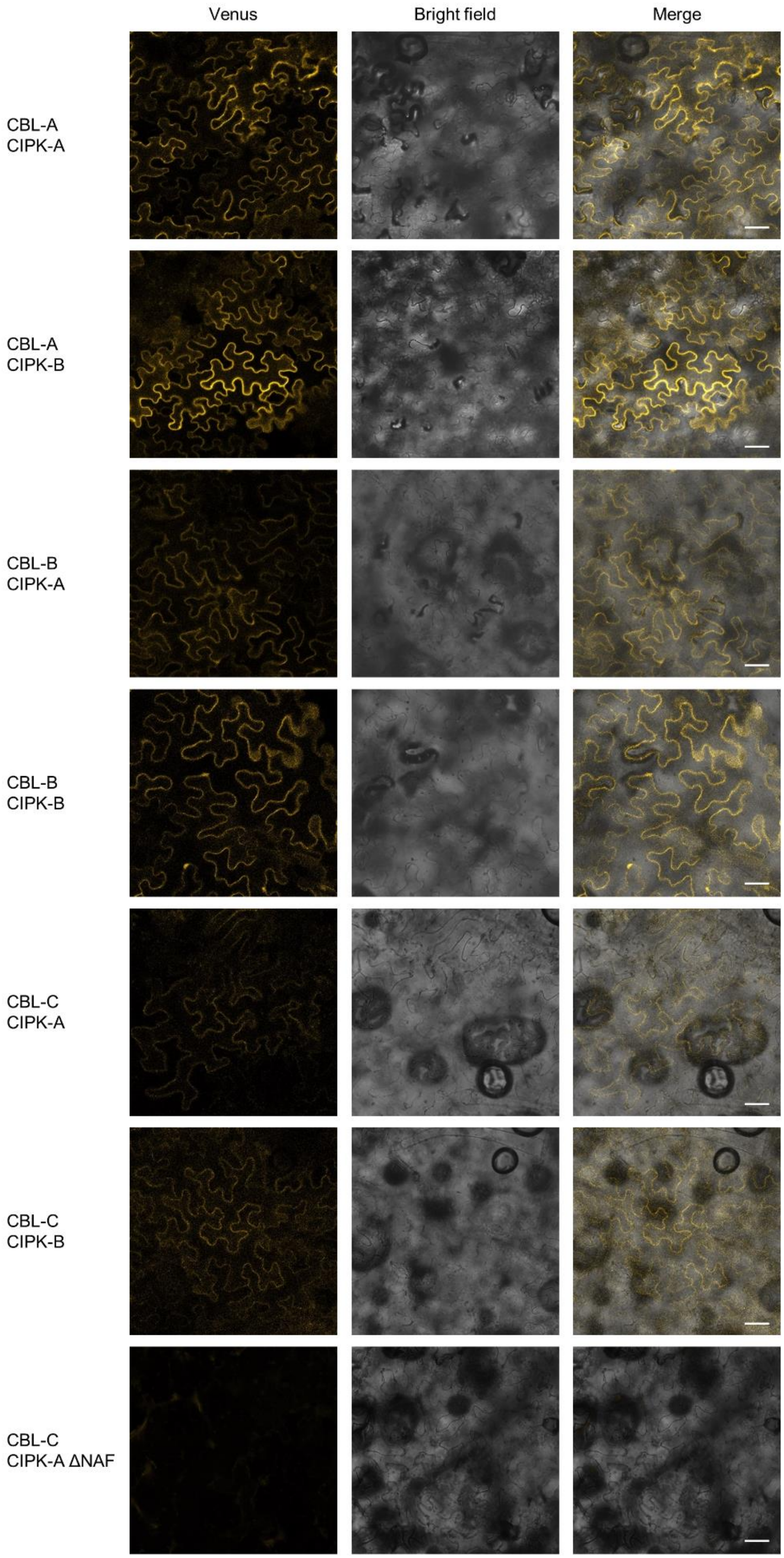
Venus
Bright field
Merge
CBL-A
CIPK-A
CBL-A
CIPK-B
CBL-B
CIPK-A
CBL-B
CIPK-B
CBL-C
CIPK-A
CBL-C
CIPK-B
CBL-C
CIPK-A ΔNAF

Fig. S5: Bimolecular fluorescence complementation confirms all *Marchantia polymorpha* CBLs and CIPKS interact in *N. benthamiana*. Constructs containing the C-terminal half of the Venus fluorescent protein fused to each CIPK (Venus^{C}-CIPK) and the N-terminal half of Venus fused to each CBL (CBL-Venus^{N}) were transiently expressed in *N. benthamiana* leaf epidermal cells. Venus fluorescence, bright field and merged images are shown for each *M. polymorpha* CBL-CIPK interaction. Scale bar in all panels = 100 µm.

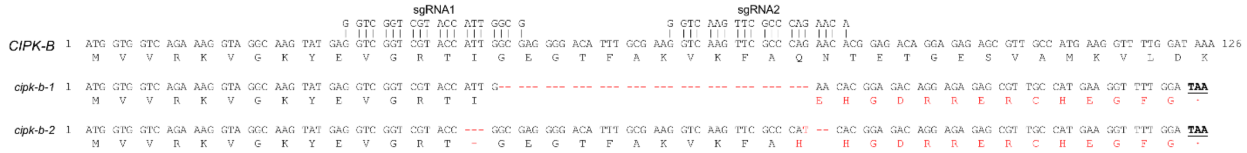

Fig. S6: Both *cipk-b* knockout mutant lines encode proteins with premature stop codons. Two 20-nucleotide sgRNAs (indicated) were designed to target exon 1 of *CIPK-B* to generate two independent *cipk-b* mutant lines via CRISPR/Cas9 gene editing in the Cam-2 background. Nucleotide deletions and substitutions in the resulting two *cipk-b* mutant lines are indicated in red. The protein sequence encoded by each mutant line is also shown, with amino acid differences compared with the wildtype CIPK-B sequence indicated in red. The *cipk-b-1* mutant contains a 35 bp deletion and the *cipk-b-2* mutant contains two small deletions and a single nucleotide substitution (Fig. 5), both leading to missense mutations and introduction of a premature stop codon (resulting in predicted proteins containing 29 and 39 amino acids, respectively).

Table S1: Gene ID numbers for sequences used in bioinformatics in this study

Gene name	Species	Gene ID
MpCBL-A	*M. polymorpha*	Mp2g07750 (Mapoly0015s0061)
MpCBL-B	*M. polymorpha*	Mp4g00900 (Mapoly0066s0053)
MpCBL-C	*M. polymorpha*	Mp5g19810 (Mapoly0134s0040)
MpCIPK-A	*M. polymorpha*	Mp1g05680 (Mapoly0005s0039)
MpCIPK-B	*M. polymorpha*	Mp2g26670 (Mapoly0025s0017)
KnCBL1	*K. nitens*	GAQ81593.1
KnCBL2	*K. nitens*	GAQ84394.1
KnCBL3	*K. nitens*	GAQ80563.1
KnCIPK1	*K. nitens*	GAQ84395.1
PpCBL1	*P. patens*	Pp3c1_36780V3.1.p
PpCBL2	*P. patens*	Pp3c16_24350V3.1.p
PpCBL3	*P. patens*	Pp3c13_3530V3.1.p
PpCBL4	*P. patens*	Pp3c5_9970V3.1.p
PpCIPK1	*P. patens*	Pp3c16_15230V3.1.p
PpCIPK2	*P. patens*	Pp3c2_13790V3.1.p
PpCIPK3	*P. patens*	Pp3c10_7160V3.1.p
PpCIPK4	*P. patens*	Pp3c14_7960V3.1.p
PpCIPK5	*P. patens*	Pp3c5_7750V3.1.p
PpCIPK6	*P. patens*	Pp3c12_210V3.1.p
PpCIPK7	*P. patens*	Pp3c15_1210V3.1.p

Table S2: Primers used for RT-qPCR in this study

Gene name	Gene ID	Forward primer	Reverse primer
CBL-A	Mp2g07750	AGCGGAAAGAGGTGAAACGG	GAGAGGGATGCTGCTGAACC
CBL-B	Mp4g00900	GGGCTGCTTCAGCTCAAAAC	CGCAAGCTGGAACTCTTCCT
CBL-C	Mp5g19810	CAAGTGCTCCACCAGAGGAC	GCCTCCGCAAATGTCTTGTC
CIPK-A	Mp1g05680	AAACACCCTGCGAACGAGAT	ACCTCAAACACCTCTGTGGC
CIPK-B	Mp2g26670	CCTGTACGGATGCACGATGA	AGAACGGAAAGGTTGAGCCC
LEA-like4	Mp1g23200	GCTAACAGACCCAGGTGAC	TGTTTCCAACGGCAGAGTG
ACT1	Mp6g10990	GAGCGCGGTTACTCTTTCAC	GACCGTCAGGAAGCTCGTAG
APT	Mp3g35140	CGAAAGCCCAAGAAGCTACC	GTACCCCCGGTTGCAATAAG

Table S3: Final constructs created and used in this study

ID	Construct details	Experiment
01044	pTRP1-LEU2-tADH1<br>pADH1-GAL4-AD-3xHA-MpCIPK-A-tADH1	Yeast two-hybrid<br>GAL4-AD-MpCIPK-A
01045	pTRP1-LEU2-tADH1<br>pADH1-GAL4-AD-3xHA-MpCIPK-B-tADH1	Yeast two-hybrid<br>GAL4-AD-MpCIPK-B
01046	pTRP1-TRP1-tADH1<br>pADH1-GAL4-BD-3xc-myc-MpCBL-A-tADH1	Yeast two-hybrid<br>GAL4-BD-MpCBL-A
01147	pTRP1-TRP1-tADH1<br>pADH1-GAL4-BD-3xc-myc-MpCBL-B-tADH1	Yeast two-hybrid<br>GAL4-BD-MpCBL-B
01048	pTRP1-TRP1-tADH1<br>pADH1-GAL4-BD-3xc-myc-MpCBL-C-tADH1	Yeast two-hybrid<br>GAL4-BD-MpCBL-C
01133	pTRP1-LEU2-tADH1<br>pADH1-GAL4-AD-3xHA-MpCIPK-A-ΔNAF-tADH1	Yeast two-hybrid<br>GAL4-AD-MpCIPK-A ΔNAF
01134	pTRP1-LEU2-tADH1<br>pADH1-GAL4-AD-3xHA-MpCIPK-B-ΔNAF-tADH1	Yeast two-hybrid<br>GAL4-AD-MpCIPK-B ΔNAF
01145	pTRP1-TRP1-tADH1<br>pADH1-GAL4-BD-3xc-myc-AtCBL4-tADH1	Yeast two-hybrid<br>GAL4-BD-AtCBL4
01146	pTRP1-LEU2-tADH1<br>pADH1-GAL4-AD-3xHA-AtCIPK24-tADH1	Yeast two-hybrid<br>GAL4-AD-AtCIPK24
01268	p35S-MpCBL-A-LgBiT-t35S<br>pNOS-SmBiT-MpCIPK-A-tNOS<br>pAtUBI10-GUS-tNOS	Split luciferase<br>MpCBL-A-LgBiT<br>SmBiT-MpCIPK-A
01269	p35S-MpCBL-B-LgBiT-t35S<br>pNOS-SmBiT-MpCIPK-A-tNOS<br>pAtUBI10-GUS-tNOS	Split luciferase<br>MpCBL-B-LgBiT<br>SmBiT-MpCIPK-A
01270	p35S-MpCBL-C-LgBiT-t35S<br>pNOS-SmBiT-MpCIPK-A-tNOS<br>pAtUBI10-GUS-tNOS	Split luciferase<br>MpCBL-C-LgBiT<br>SmBiT-MpCIPK-A
01271	p35S-AtCBL4-LgBiT-t35S<br>pNOS-SmBiT-MpCIPK-A-tNOS<br>pAtUBI10-GUS-tNOS	Split luciferase<br>AtCBL4-LgBiT<br>SmBiT-MpCIPK-A
01272	p35S-MpCBL-A-LgBiT-t35S<br>pNOS-SmBiT-MpCIPK-B-tNOS<br>pAtUBI10-GUS-tNOS	Split luciferase<br>MpCBL-A-LgBiT<br>SmBiT-MpCIPK-B
01273	p35S-MpCBL-B-LgBiT-t35S<br>pNOS-SmBiT-MpCIPK-B-tNOS<br>pAtUBI10-GUS-tNOS	Split luciferase<br>MpCBL-B-LgBiT<br>SmBiT-MpCIPK-B
01274	p35S-MpCBL-C-LgBiT-t35S<br>pNOS-SmBiT-MpCIPK-B-tNOS<br>pAtUBI10-GUS-tNOS	Split luciferase<br>MpCBL-C-LgBiT<br>SmBiT-MpCIPK-B
01275	p35S-AtCBL4-LgBiT-t35S<br>pNOS-SmBiT-MpCIPK-B-tNOS	Split luciferase<br>AtCBL4-LgBiT

	pAtUBI10-GUS-tNOS	SmBiT-MpCIPK-B
01276	p35S-MpCBL-A-LgBiT-t35S<br>pNOS-SmBiT-MpCIPK-A-ΔNAF-tNOS<br>pAtUBI10-GUS-tNOS	Split luciferase<br>MpCBL-A-LgBiT<br>SmBiT-MpCIPK-A<br>ΔNAF
01277	p35S-MpCBL-B-LgBiT-t35S<br>pNOS-SmBiT-MpCIPK-A-ΔNAF-tNOS<br>pAtUBI10-GUS-tNOS	Split luciferase<br>MpCBL-B-LgBiT<br>SmBiT-MpCIPK-A<br>ΔNAF
01278	p35S-MpCBL-C-LgBiT-t35S<br>pNOS-SmBiT-MpCIPK-A-ΔNAF-tNOS<br>pAtUBI10-GUS-tNOS	Split luciferase<br>MpCBL-C-LgBiT<br>SmBiT-MpCIPK-A<br>ΔNAF
01279	p35S-AtCBL4-LgBiT-t35S<br>pNOS-SmBiT-MpCIPK-A-ΔNAF-tNOS<br>pAtUBI10-GUS-tNOS	Split luciferase<br>AtCBL4-LgBiT<br>SmBiT-MpCIPK-A<br>ΔNAF
01280	p35S-MpCBL-A-LgBiT-t35S<br>pNOS-SmBiT-MpCIPK-B-ΔNAF-tNOS<br>pAtUBI10-GUS-tNOS	Split luciferase<br>MpCBL-A-LgBiT<br>SmBiT-MpCIPK-B<br>ΔNAF
01281	p35S-MpCBL-B-LgBiT-t35S<br>pNOS-SmBiT-MpCIPK-B-ΔNAF-tNOS<br>pAtUBI10-GUS-tNOS	Split luciferase<br>MpCBL-B-LgBiT<br>SmBiT-MpCIPK-B<br>ΔNAF
01282	p35S-MpCBL-C-LgBiT-t35S<br>pNOS-SmBiT-MpCIPK-B-ΔNAF-tNOS<br>pAtUBI10-GUS-tNOS	Split luciferase<br>MpCBL-C-LgBiT<br>SmBiT-MpCIPK-B<br>ΔNAF
01283	p35S-AtCBL4-LgBiT-t35S<br>pNOS-SmBiT-MpCIPK-B-ΔNAF-tNOS<br>pAtUBI10-GUS-tNOS	Split luciferase<br>AtCBL4-LgBiT<br>SmBiT-MpCIPK-B<br>ΔNAF
01284	p35S-MpCBL-A-LgBiT-t35S<br>pNOS-SmBiT-AtCIPK24-tNOS<br>pAtUBI10-GUS-tNOS	Split luciferase<br>MpCBL-A-LgBiT<br>SmBiT-AtCIPK24
01285	p35S-MpCBL-B-LgBiT-t35S<br>pNOS-SmBiT-AtCIPK24-tNOS<br>pAtUBI10-GUS-tNOS	Split luciferase<br>MpCBL-B-LgBiT<br>SmBiT-AtCIPK24
01286	p35S-MpCBL-C-LgBiT-t35S<br>pNOS-SmBiT-AtCIPK24-tNOS<br>pAtUBI10-GUS-tNOS	Split luciferase<br>MpCBL-C-LgBiT<br>SmBiT-AtCIPK24
01287	p35S-AtCBL4-LgBiT-t35S<br>pNOS-SmBiT-AtCIPK24-A-tNOS<br>pAtUBI10-GUS-tNOS	Split luciferase<br>AtCBL4-LgBiT<br>SmBiT-AtCIPK24
01288	p35S-MpCBL-A-LgBiT-t35S	Split luciferase

	pNOS-LgBiT-MpCIPK-A-tNOS<br>pAtUBI10-GUS-tNOS	MpCBL-A-LgBiT<br>LgBiT-MpCIPK-A
01289	p35S-MpCBL-A-SmBiT-t35S<br>pNOS-SmBiT-MpCIPK-A-tNOS<br>pAtUBI10-GUS-tNOS	Split luciferase<br>MpCBL-A-SmBiT<br>SmBiT-MpCIPK-A
01247	p35S-HYG-tNOS<br>pMpEF1α-NLS-pcoCas9-t35S<br>pMpU6-1-sgRNA-MpCIPK-B1-tRNAP<br>pMpU6-1-sgRNA-MpCIPK-B2-tRNAP	CRISPR/Cas9<br>MpCIPK-B knockout
01135	pNOS-KAN-tNOS<br>p35S-MpCBL-A-VYNE-t35S<br>pAtUBI10-dsRED-tNOS	BiFC<br>MpCBL-A-Venus^{N}
01188	pNOS-KAN-tNOS<br>p35S-MpCBL-B-VYNE-t35S<br>pAtUBI10-dsRED-tNOS	BiFC<br>MpCBL-B-Venus^{N}
01137	pNOS-KAN-tNOS<br>p35S-MpCBL-C-VYNE-t35S<br>pAtUBI10-dsRED-tNOS	BiFC<br>MpCBL-C-Venus^{N}
01138	pNOS-KAN-tNOS<br>p35S-VYCE(R)-MpCIPK-A-t35S<br>pAtUBI10-dsRED-tNOS	BiFC<br>Venus^{C}-MpCIPK-A
01139	pNOS-KAN-tNOS<br>p35S-VYCE(R)-MpCIPK-B-t35S<br>pAtUBI10-dsRED-tNOS	BiFC<br>Venus^{C}-MpCIPK-B
01140	pNOS-KAN-tNOS<br>p35S-VYCE(R)-MpCIPK-A-ΔNAF-t35S<br>pAtUBI10-dsRED-tNOS	BiFC<br>Venus^{C}-MpCIPK-A<br>ΔNAF
